# Biomechanically Informed Image Registration for Patient-Specific Aortic Valve Strain Analysis

**Published:** 2026-01-07

**Authors:** Mohsen Nakhaei, Alison Pouch, Silvani Amin, Matthew Daemer, Christian Herz, Natalie Yushkevich, Lourdes Al Ghofaily, Nimesh Desai, Joseph Bavaria, Matthew Jolley, Wensi Wu

**Affiliations:** a Department of Anesthesiology and Critical Care Medicine, Children’s Hospital of Philadelphia, Philadelphia, PA, USA; b Department of Radiology and Bioengineering, University of Pennsylvania, Philadelphia, PA, USA; c Department of Anesthesiology, University of Pennsylvania, University of Pennsylvania, Philadelphia, PA, USA; d Department of surgery, University of Pennsylvania, Philadelphia, PA, USA; e Department of cardiac surgery, Jefferson Health, Philadelphia, PA, USA; f Department of Mechanical Engineering and Applied Mechanics, University of Pennsylvania, Philadelphia, PA, USA; g Cardiovascular Institute, Children’s Hospital of Philadelphia, Philadelphia, PA, USA

**Keywords:** Aortic valve biomechanics, Finite element simulation, Image registration, Computational biomechanics

## Abstract

Aortic valve (AV) biomechanics play a critical role in maintaining normal cardiac function. Pathological variations, particularly in bicuspid aortic valves (BAVs), alter leaflet loading, increase strain, and accelerate disease progression. Accurate, patient-specific characterization of valve geometry and deformation is essential for predicting disease progression and guiding durable repair. Current imaging and computational methods often fail to capture rapid valve motion and complex patient-specific features. To address these challenges, we combined image registration with finite element method (FEM) to enhance AV tracking and biomechanical assessment. Patient-specific valve geometries from 4D transesophageal echocardiography (TEE) and CT were used in FEM to model AV closure and generate intermediate deformation states. The FEM-generated states facilitated leaflet tracking, while the registration algorithm corrected mismatches between simulation and image. Across 20 patients, FEM-augmented registration improved accuracy by 40% compared with direct registration (33% for TEE, 46% for CT). This improved tracking enabled more reliable strain estimation by measuring leaflet deformation directly from imaging and reducing uncertainties from boundary conditions (i.e., constraints and loading) and material assumptions. Areal and Green-Lagrange strains, as well as effective (deviatoric) strain, were quantified in adult trileaflet/bicuspid, and pediatric patients. Trileaflet adults showed uniform deformation, BAVs exhibited asymmetric strain, and pediatric valves had low mean areal strain with high variability. Convergence between trileaflet adult and pediatric valves in mean effective strain suggests volumetric deformation drives age- and size-related differences. The FEM-augmented registration framework enhances geometric tracking, improves biomechanical accuracy, and provides clinically relevant insights into patient-specific AV deformation, supporting individualized intervention planning.

## 1. Introduction

The aortic valve (AV) plays an essential role in cardiovascular function by regulating blood flow from the left ventricle to the aorta and preventing regurgitation during diastole. In healthy individuals, the trileaflet aortic valve (TAV) consists of three leaflets that undergo large, rapid, and highly anisotropic deformations throughout each cardiac cycle. These deformations expose valvular interstitial cells (VICs) to cyclic strains that regulate extracellular matrix (ECM) and maintain tissue homeostasis (West et al., 2023; Huang et al., 2007; Yap et al., 2010; Ayoub et al., 2017). Strain thus serves as the critical mechanobiological link connecting valve structure and the hemodynamic forces imposed on the valve to cellular behavior and long-term tissue remodeling. When strain patterns become abnormal (e.g., due to age-related degeneration, altered hemodynamics, or congenital defects), VICs activation and ECM dysregulation accelerate disease progression. A prominent case of this is calcific aortic valve disease (CAVD) (Davies et al., 1996; Yap et al., 2010; Ayoub et al., 2017, 2021; Lam et al., 2016; Decano et al., 2022; Aggarwal et al., 2014).

Among congenital defects, bicuspid aortic valve (BAV) provides a clear example of how altered strain drives pathology. BAV, affecting 1–2% of the population (Evangelista, 2011), results from fusion of adjacent leaflets, producing asymmetric geometry and disordered loading. These structural changes elevate localized stresses and strains, particularly near the raphe (Jermihov et al., 2011; Merritt et al., 2014; Rego et al., 2022). These mechanical abnormalities contribute to early ECM disorganization, rapid calcification, and accelerated onset of aortic stenosis (AS) or regurgitation (AR), often decades earlier than in tricuspid valves (Rutkovskiy et al., 2017; Khang et al., 2023). Given that strain may play a central role in regulating cellular responses and long-term tissue remodeling, accurate patient-specific quantification of leaflet strain is critical. Such measurements provide valuable insight into disease progression and help identify tissue regions at highest risk for failure. Although not yet part of routine surgical practice, these strain-based assessments have the potential to inform personalized procedural planning (such as cusp reconstruction or prosthetic valve design) by guiding more durable repairs that facilitate optimal tissue performance, ultimately reducing the need for repeat interventions.

Despite this clear clinical need for precise strain assessment, in vivo strain estimation remains a major unsolved challenge. Clinical imaging modalities, including 4D computed tomography (CT) and 4D echocardiography, do not acquire enough frames during the extremely rapid transition between valve opening and closure. These abrupt geometric changes exceed both the temporal and spatial resolution of current imaging systems (Meredith et al., 2025). Because conventional image-based registration algorithms fundamentally rely on incremental motion between sequential frames, the sparse sampling during fast deformation phases leads to large tracking errors. Additionally, limited soft tissue contrast and acoustic shadowing further degrade the accuracy of deformation estimates (Meredith et al., 2025). As a result, existing registration approaches struggle to propagate leaflet geometry across the full cardiac cycle and cannot reliably compute physiologically meaningful strain fields.

Since image-based tracking methods cannot recover full-cycle deformation, finite element (FE) and fluid-structure interaction (FSI) modeling provide complementary advantages and offer a potential path to fill this gap, although these methods still face significant limitations. Prior computational studies have demonstrated important insights regarding leaflet mechanics and hemodynamics (Labrosse et al., 2015; Chen et al., 2022; Lior et al., 2023; Jermihov et al., 2011; Rego et al., 2022; Marom et al., 2012; Yin et al., 2024). However, most models rely on idealized geometries, such as the simplified BAV geometry in a FE model used by Jermihov et al. (2011) or the parametric leaflet surfaces used in full-cycle FSI simulations (Yin et al., 2024). Many also depend on estimated or assumed material properties, as in the 4D transesophageal echocardiography (TEE) based modeling by Labrosse et al. (2015), and simplified boundary conditions (BCs), as in Marom et al. (2012). High computational cost and uncertain loading definitions further limit model accuracy, and few approaches have undergone robust clinical validation, with several demonstrated only in small samples or single subjects (Labrosse et al., 2015; Yin et al., 2024). Collectively, these constraints limit the ability of existing forward FE and FSI frameworks to reconstruct truly patient-specific valve deformation.

While forward FE and FSI simulations can estimate full-cycle valve deformation, they alone cannot fully resolve challenges posed by patient-specific variability, including differences in geometry, material properties, and boundary conditions. Image-based tracking alone cannot capture the rapid movement of leaflets, and existing FE models often rely on idealized geometries or estimated material properties, which limits their accuracy. To bridge these complementary gaps, FE simulation can be leveraged to generate mechanically consistent intermediate deformation states that are missing from sparse imaging sequences, enabling direct computation of stresses and strains across the full cardiac cycle (Wu et al., 2024). Building on this idea, we introduced a patient-specific computational framework that couples finite element simulation with deformable image registration. The FE model provides biomechanically informed intermediate configurations, facilitating robust alignment between anatomically distant structures at two time frames, such as fully open and fully closed valves. Image registration, in turn, corrects deviations arising from modeling assumptions and ensures that leaflet geometry accurately reflects the patient-specific imaging data. By combining the strengths of mechanics-based modeling and image-based registration, this framework enables robust tracking of aortic valve deformation and accurate computation of leaflet strains, overcoming the fundamental limitations of imaging-only and simulation-only approaches. We further evaluated registration accuracy and computed strain distributions across different imaging modalities, including 4D CT and 4D TEE, and across multiple patient cases, including adult trileaflet, bicuspid, and pediatric aortic valves. Ultimately, this work establishes a new pathway for precise patient-specific biomechanical assessment, enabling quantification of leaflet strains that can directly inform tissue-level risk and disease progression.

## 2. Methods

**Figure 1:**
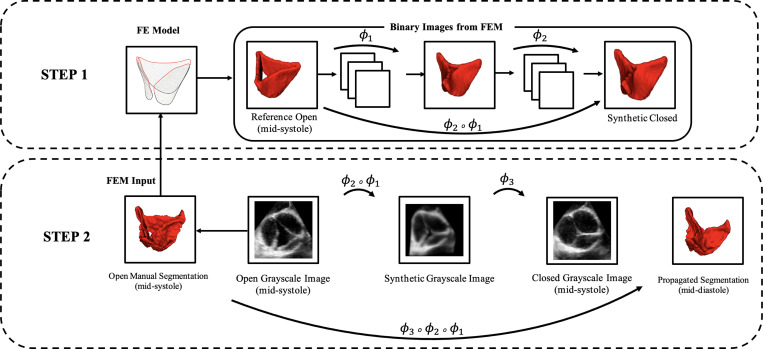
Registration pipeline with binary images from FE simulation.

Conventional registration methods cannot accurately align the aortic valve between its two extreme configurations, mid-systolic (AV open) and mid-diastolic (AV closed), because the valve’s complex geometry undergoes rapid movement, substantial non-linear deformation, and significant shape changes throughout the cardiac cycle. As a result, conventional feature-tracking approaches have difficulty obtaining anatomically consistent models throughout the cardiac phases. To address this limitation, we proposed a hybrid framework that integrated biomechanical simulation using an FE model with image-based registration, as shown in Figure 1.

The process begins with the manual segmentation of the aortic valve in the mid-systole frame (open reference configuration). In the first step, the medial surface of the valve leaflets is extracted from the mid-systole segmentation (AV open) and used to generate a finite element model to simulate valve closure. To characterize the biomechanical behavior of the valve tissue under physiological loading, material properties and diastolic pressure values were obtained from literature and the patient database (when available). Furthermore, physiologically informed boundary conditions obtained from direct registration and were applied to the leaflets to simulate the valve’s deformation from the mid-systole configuration (AV open) to the mid-diastole configuration (AV closed). The simulation generates a sequence of intermediate geometries that approximate the valve’s movement between cardiac phases, from the mid-systolic configuration to a closed configuration near mid-diastole, which we refer to as the “synthetic closed” state. In the second step, these intermediate geometries serve as prior knowledge within the registration pipeline, guiding the propagation of mid-systolic segmentation across 4D TEE or 4D CT images. As a result, we obtain an image-driven model of the aortic valve and can evaluate the strain throughout the cardiac phases. In the following, we go through these steps in detail.

### 2.1. Model preparation from clinical images

#### 2.1.1. Segmentation

An accurate geometric representation of the aortic valve is a crucial step in developing a physiologically relevant computational model for biomechanical simulation and deformation analysis. In this study, manual segmentation was used to extract the aortic valve anatomy from 4D TEE or 4D CT images acquired throughout the cardiac cycle. In total, we curated 14 4D TEE datasets and 6 4D CT datasets, 4 of which were from pediatric patients. The collection and analysis of these data were approved by the Institutional Review Boards at the Children’s Hospital of Philadelphia and the University of Pennsylvania. Two specific frames were selected for segmentation: (1) the mid-systolic frame, representing the fully open configuration of the valve, and (2) the mid-diastolic frame, representing the fully closed configuration. These frames capture the two extremes of valve deformation during cardiac cycle and provide crucial reference states for both simulation and registration.

Manual segmentation was performed using ITK-SNAP and 3D Slicer, two widely used software platforms for anatomical image labeling (Yushkevich et al., 2006; Fedorov et al., 2012).

Although ML-based segmentation methods have demonstrated good performance in many cardiac imaging applications, their accuracy depends heavily on image characteristics and on training data that reflect similar anatomical features and acquisition settings. To ensure the highest level of accuracy and consistency in the geometric models used for our analysis, we elected to perform manual segmentation for all patients.

Segmentation focused on labeling the individual leaflets and the 3D annular contour in both frames. The open-frame segmentation served as input for extracting the leaflet surface and developing the FE model, while the closed-frame segmentation served as a reference or “ground truth” for validating propagated segmentations. Because the spatial resolution was limited by the original image acquisition, careful attention was required during segmentation to preserve anatomical details. To preserve key morphological features such as leaflet curvature, commissures, and attachment regions, each frame was manually traced slice by slice across multiple 2D planes, carefully reviewed in orthogonal views, and processed with minimal smoothing to keep small anatomical details accurate. Label maps were then interpolated into 3D volumes, with anatomical landmarks such as the valve annulus and sinus borders guiding the contours to maintain consistency across the two frames. Segmentation was performed under the supervision of a clinical expert to reduce operator bias and ensure accurate representation of the native leaflet anatomy for physiologically realistic simulations. The final label maps were converted into surface meshes for further modeling steps.

#### 2.1.2. Medial surface extraction

**Figure 2:**
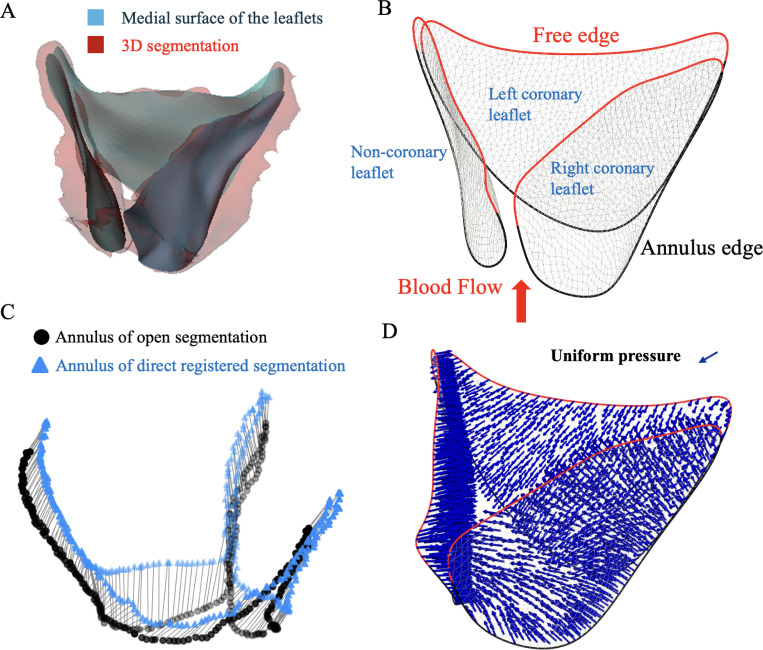
Finite element model development. (A) Extraction of medial surface of the valve leaflets at mid-systole. (B) Definition of boundary conditions for the annulus and the free edge of the leaflet. (D) Estimation of annulus displacement. (C) Imposed uniform diastolic pressure on the leaflets.

After segmenting the valve in the mid-systolic (open) configuration, the medial surface was extracted from the segmentation to serve as the input geometry for FE analysis (Figure 2 (A)). The medial surface, representing the mid-surface of the valve leaflets, was generated using an interactive skeletonization approach applied to the surface mesh of the binary segmented volume, implemented in 3D Slicer (Herz et al., 2024). For completely fused leaflets, the two leaflets were modeled as a single entity to maintain geometric consistency in the medial surface representation.

The medial surface reduces the computational complexity of the FE model while preserving essential anatomical features such as leaflet curvature, commissures, the annular boundary, and the free edges. It also provides a mid-surface reference, from which thickness profiles can be defined across the leaflet to generate shell elements that accurately reflect the leaflet’s 3D geometry and mechanical behavior. It is important to note that for the fused leaflet we modeled the two leaflets as one.

Post-processing of the medial surface included smoothing with preserved boundaries, which were carried out using the FEBio software (Maas et al., 2012). The result was a continuous, smooth 3D surface that accurately captured the valve geometry in the open reference configuration. This process ensures that the mesh topology is suitable for finite element simulation. This surface was later used to generate a shell-based finite element model, which serves as the initial configuration in simulating valve closure (Figure 2 (B)).

### 2.2. Finite element valve model

#### 2.2.1. Constitutive model

The non-linear, nearly-incompressible mechanical behavior of aortic valve tissue was modeled using a hyperelastic constitutive model. Specifically, we employed the isotropic Lee–Sacks model, a Fung-type exponential strain-energy function previously developed for heart valve simulations (Sun and Sacks, 2005; Fan and Sacks, 2014). This model combines a neo-Hookean matrix with a Fung-type exponential term to capture the nonlinear response of soft biological tissues under large deformations. In addition, it separates the contributions of isochoric deformations and volumetric deformations, allowing the energy associated with shape changes $\left({\text{Ψ}}_{\text{isochoric}}\right)$ and volume changes $\left(U_{vol}\right)$ to be considered independently. The total strain-energy density can be expressed as,

(1) $$\text{Ψ}(C,J)={\text{Ψ}}_{\text{isochoric}}\left(c_{0},c_{1},c_{2},I_{1}\right)+U_{vol}(J)$$

where the isochoric strain-energy density defined as,

(2) $${\text{Ψ}}_{\text{isochoric}}=\frac{c_{0}}{2}\left(I_{1}-3\right)+\frac{c_{1}}{2}\left(e^{c_{2}{\left(I_{1}-3\right)}^{2}}-1\right).$$

C is the right Cauchy-Green deformation tensor representing the full deformation, $I_{1}=\mathrm{tr}(C)$ is its first invariant, and J=det(F) denotes the local volumetric change. The parameter $c_{0}$ represents the shear modulus of the neo-Hookean matrix, which governs the baseline linear elasticity of the tissue. The parameters $c_{1}$ and $c_{2}$ control the magnitude and rate of exponential stiffening, respectively, and are key to capturing the nonlinear behavior of collagenous soft tissues.

For these materials, the entire bulk (volumetric) behavior is determined by the function $U_{vol}(J)$. This function is constructed to have a value of 0 for J=1 and to be positive for all other values of J>0. In quadratic form, it can be written as $U_{vol}=\frac{k}{2}{\left(J-1\right)}^{2}$, where k is the bulk modulus, controlling the tissue’s resistance to volumetric changes and ensuring near-incompressibility, which is a common property of biological soft tissues.

The material parameters used in this study were k=5000 kPa, $c_{0}=67$ kPa, $c_{1}=13$ kPa, and $c_{2}=35$. These values were chosen based on prior studies on material properties of heart valves (Wu et al., 2018), where parameters of the Lee–Sacks material model were determined by fitting equibiaxial experimental data reported in Sun and Sacks (2005); Lee et al. (2014).

While the Fung-type model offers realistic mechanical behavior, it is known to introduce convergence challenges under certain loading conditions. Kamensky et al. (2018) reported that the exponential term may cause these convergence difficulties, and therefore, they introduced a tangent scale factor which can improve numerical stability. In our simulations, a tangent scale factor of one was applied, but model convergence was monitored carefully to mitigate any divergence issues.

Although recent studies such as Armfield et al. (2024) have highlighted the importance of anisotropic behavior in valve leaflets due to collagen fiber alignment, we assumed isotropic material behavior for the sake of simplicity. It is acknowledged that this assumption introduces some degree of approximation, particularly in commissural regions where fiber orientation affects local deformation. However, in this study, the FE simulation is not intended to predict exact valve deformation but rather to generate intermediate valve configurations that serve as a structural prior for guiding the registration process. We assume that minor inaccuracies introduced by the isotropic assumption and uniform material properties for all the leaflets are compensated during the image-based registration step.

#### 2.2.2. Boundary conditions and loading

Physiological boundary conditions (annulus movement and pressure loading on the valve leaflets) were applied to the reference open valve shell model to approximate the nearly closed configuration, referred to as the “synthetic closed” state. In particular, the annulus displacement during the cardiac phases was applied to the annulus to capture the dynamics of the aortic root. This displacement was obtained in a patient-specific manner from initial direct image registration. First, we directly registered the open mid-systolic grayscale image into the mid-diastolic image and we propagated the open segmentation in mid-systolic accordingly to obtain the segmentation in mid-diastolic frame. Therefore, we can approximate the annulus displacement between the two configurations (Figure 2 (C)) and impose it in our FE simulation. The free edges of the valve leaflets were left unconstrained.

Furthermore, pressure loading on the leaflets was applied to simulate diastolic closure. The transvalvular pressure gradient for AV, defined as the pressure difference between the left ventricle (LV) and the aorta (AO), $\left(P=P_{LV}-P_{AO}\right)$, was reported over a cardiac cycle of 0.76 s (Kim et al., 2008; Hole et al., 1996). They reported that during the opening phase (t=0 s to t=0.18 s), only a small pressure acted on the ventricular side of the leaflets, decreasing from about 4 mmHg to 0 mmHg. Once the closing phase began, pressure was applied on the aortic side and increased sharply between t=0.23 s and t=0.3 s, reaching approximately 80–100 mmHg at its peak. However, the actual pressure experienced by the leaflets during diastole is lower than systemic aortic diastolic pressure. Factors contributing to this include: (i) the blood in the aortic sinuses moves in small circular patterns that create a soft fluid cushion (fluid cushioning), so the leaflets do not feel the full aortic pressure (Marom et al., 2012; ?); (ii) leaflet geometry and coaptation mechanics, which create non-uniform load distribution; and (iii) patient-specific hemodynamics, notably in aortic regurgitation (AR), where retrograde flow during diastole reduces local pressure near the leaflets (Yang et al., 2020). Clinical data show that adults with moderate-to-severe AR often have lower diastolic pressures, illustrating inter-patient variability (Yang et al., 2020).

Simplified FE models applying uniform pressure overestimate leaflet stress compared to fully coupled FSI simulations (Marom et al., 2012). Assuming a typical systemic diastolic pressure of approximately 80 mmHg in adults, and considering that FSI studies suggest only about 90–95% of this pressure is effectively transmitted to the leaflets due to fluid cushioning and coaptation dynamics (Marom et al., 2012), we selected a uniform aortic-side pressure of 75 mmHg (≈ 94%) to balance physiological realism, patient variability, and computational feasibility for all 16 adult cases (Figure 2 (D)). For the pediatric patient, where diastolic blood pressure measured before CT was available, an average diastolic pressure of 45 mmHg was applied. It is important to note that the transvalvular pressure gradient reflects how hard the heart must work to push blood through the aortic valve and varies among patients depending on the severity of aortic stenosis or regurgitation (Khalili et al., 2022). Accordingly, the subsequent image registration step corrects small geometric deviations that may arise from applying a uniform pressure, ensuring that the resulting leaflet configuration remains consistent with the patient-specific imaging data.

#### 2.2.3. Simulation and model convergence

The finite element model was solved using the dynamic implicit solver of FEBio (Maas et al., 2012). The valve leaflets were modeled using linear triangular shell elements with a uniform thickness of 1.2 mm for adult patients and 1 mm for pediatric patients. The thickness value for aortic valve was obtained from Sahasakul et al. (1988), where they reported mean thicknesses for aortic valve for different age groups. The simulation was performed multiple times with different mesh densities to assess convergence behavior. Mesh sizes were systematically reduced, and with a target edge length of 0.3 mm for the triangular elements, convergence in displacement was achieved. The simulation generated a series of intermediate frames between the fully open and fully closed states, and approximated the “synthetic closed” configuration, which was exported to guide registration.

### 2.3. Image registration

Standard image-registration methods struggle with the large deformation of the aortic valve between systole and diastole, leading to misaligned propagated segmentations. To address this, we developed a biomechanically informed registration framework to improve robustness in tracking the aortic valve feature across cardiac phases. First, FE simulations generate a sequence of intermediate valve configurations as binary images; second, these intermediate frames are used to guide the registration (see Figure 1).

In the first step, given the open manual segmentation at mid-systole, we develop a medial surface of the valve (Herz et al., 2024) and simulate valve closure. This simulation brings the aortic valve toward its mid-diastolic configuration, which we refer to as the “synthetic closed” state. From the FE simulation, we extract three frames: the initial, middle, and the final simulation frame, and convert these into binary images. These binary images are then resliced and resampled into the grayscale image space. Then, pairwise registration between the frames is performed to compute the transformations that map one configuration to the next. The composed transformation $\left(\phi_{2}\circ \phi_{1}\right)$ therefore maps the mid-systolic open segmentation to the synthetic closed configuration.

In the second step, this FE-derived transformation $\left(\phi_{2}\circ \phi_{1}\right)$ is applied to the mid-systolic grayscale image to obtain a synthetic closed image that closely approximates the actual mid-diastolic frame. This facilitates the final registration from the synthetic closed to the actual closed grayscale image in mid-diastole $\left(\phi_{3}\right)$. The full composed transformation $\left(\phi_{3}\circ \phi_{2}\circ \phi_{1}\right)$ is then used to propagate the manual open segmentation from mid-systole into the closed configuration at mid-diastole.

The registration was performed between the FE frames using affine and deformable registration with Greedy (Yushkevich et al., 2016) to obtain a composed transformation mapping the open configuration to the closed configuration. For affine registration, Greedy optimized an affine transformation matrix, initialized by matching the centers of the fixed and moving images (-ia-image-centers), using a sum of squared differences (SSD) metric for segmentation and a normalized cross-correlation (NCC) metric for grayscale images. The NCC metric used a local kernel (radius 2 voxels), and the registration employed a multi-resolution schedule of 100×50×10 iterations across three resolution levels (coarse, intermediate and full resolution). For deformable registration, Greedy computed a dense displacement field (warp), initialized from the affine result (-it affine.mat), optimized using the same multi-resolution schedule, SSD or NCC depending on the image type, and a stationary velocity field model with Lie bracket correction (-svlb) which applies a more precise update to the velocity field, improving the accuracy of the resulting deformations. The computed warp and affine transformations were then used to resample both grayscale images and segmentation maps into the fixed frame, with label interpolation (-ri LABEL 0.2vox) applied to segmentations to minimize artifacts along boundaries.

This registration process ensures that the propagated segmentation remains aligned with patient anatomical images and eliminates the errors that could arise from simulation assumptions. The goal is to enable the construction of a precise, image-driven valve model throughout the cardiac phases.

### 2.4. Strain calculation

We compute the total strain on the leaflet for each subject by summing the strains from both steps. For the first step, we compute both the areal strain and the Green–Lagrange strain at the synthetic closed configuration obtained from the FE simulation. The areal strain for each triangular element in the FE mesh is defined as the relative change in its surface area between two configurations,

(3) $$\varepsilon_{A}=\frac{A_{\text{d}}-A_{\text{r}}}{A_{\text{r}}},$$

where $A_{\text{r}}$ is the area of the element in the reference configuration (open manual segmentation) and $A_{\text{d}}$ is the area of the same element in the deformed configuration (synthetic closed). For the second step, we compute the deformation between the synthetic closed and the closed configuration. The total areal strain is obtained as the sum of the areal strain components from both steps,

(4) $$\varepsilon_{A,\text{total}}=\varepsilon_{A,1}+\varepsilon_{A,2}.$$

The Green-Lagrange strain tensor E is computed from the deformation gradient as,

(5) $$E=\frac{1}{2}\left(F^{\text{T}}F-I\right),$$

where F is the deformation gradient computed within the FE solver, and I is the identity tensor. However, to compute the Green–Lagrange strain in the second step, we calculate the in-plane Green–Lagrange strain directly from the nodal coordinates of the shell meshes in the two configurations. In the first step, the FE simulation deforms the leaflets from the open configuration to a near-closed state at mid-diastole, during which most of the deformation occurs, particularly out-of-plane bending. In the second step, the leaflets are already closed, and only small deformations, mainly in-plane, arise from the registration process, which corrects the leaflet geometry and aligns it with the patient-specific anatomy. Because the deformations in the second step are small, it is reasonable to approximate the strain as in-plane, and this assumption is verified. Finally, the total Green–Lagrange strain is obtained by summing the FE-derived Green–Lagrange strain from the first step with the in-plane strain from the second step,

$$E_{\text{total}}=E_{\text{FE}}+E_{2D}^{\left(3D\right)},$$

where,

$$E_{2D}^{\left(3D\right)}=\left[\begin{array}{ccc} E_{xx} & E_{xy} & 0\\ E_{xy} & E_{yy} & 0\\ 0 & 0 & 0 \end{array}\right].$$

The effective (von Mises–type) strain, a scalar measure of deformation, is computed from the total strain tensor $E_{\text{total}}$ as,

$$\varepsilon_{\text{eff}}=\sqrt{E^{\prime }:E^{\prime }},$$

where,

$$E^{\prime }=E_{\text{total}}-\frac{1}{3}\text{tr}\left(E_{\text{total}}\right)I,$$

is the deviatoric part of the strain tensor. In addition, the Green–Lagrange strain magnitude, which provides an overall measure of local deformation including volumetric changes, is defined as

$$\varepsilon_{\text{GL}}=\sqrt{E_{\text{total}}:E_{\text{total}}}.$$

Together, these scalar measures allow quantification of both deviatoric (shape-changing) and total (overall) deformation in the aortic valve tissue.

Finally, strain distributions were visualized using colormaps overlaid on the medial surface mesh, enabling the identification of regions of elevated strain, particularly near the commissures and coaptation lines. These results provide insights into the biomechanical behavior of the valve, support the assessment of pathological conditions such as leaflet stiffening or prolapse, and could potentially guide strategies for valve repair surgery.

## 3. Results

We applied the methodology to 20 patients (16 adult and 4 pediatric), including 14 4D TEE and 6 4D CT datasets. These images were acquired intraoperatively or immediately prior to the procedure. Patient characteristics, including valve morphology and severity of aortic regurgitation (AR) and stenosis (AS), are summarized in Table 1. Overall, the combined cohort represented a broad spectrum of valve morphologies and disease severities, encompassing patients with normal to severely regurgitant valves and none to moderate stenosis.

**Figure 3:**
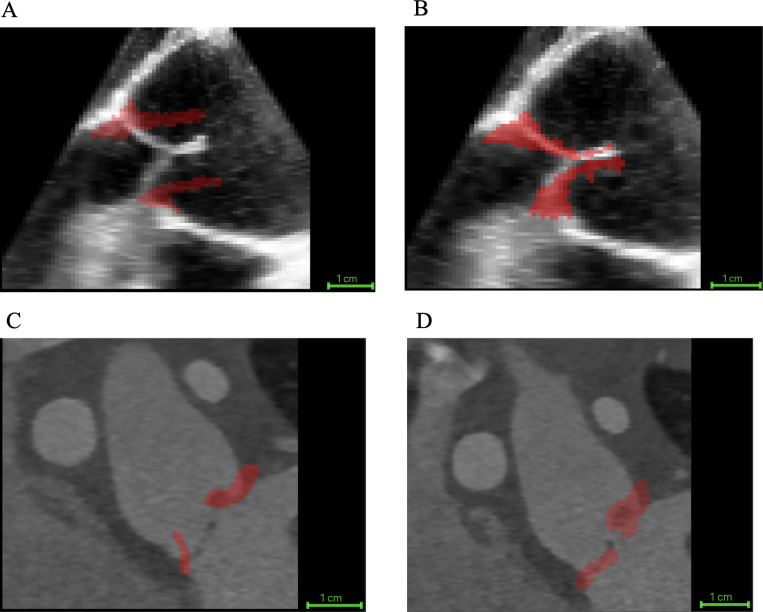
Example of image-based registration results for one TEE case and one CT case. The reconstructed aortic valve leaflets at mid-diastole are shown in red. (A) TEE with direct registration, (B) TEE with FEM-augmented registration, (C) CT with direct registration, and (D) CT with FEM-augmented registration.

The proposed biomechanically informed registration improved segmentation-tracking accuracy by an average of 40%, reflecting the reduction in the mean distance between the tracked closed-state segmentation and the manual ground truth across all 20 cases when compared with direct registration. Specifically, this mean distance decreased from 3.70 ± 2.30 mm with direct registration (no FEM) to 2.23 ± 1.27 mm using our approach. Figure 3 illustrates an example of the reconstructed patient-specific aortic valve closure (shown in red) overlaid on mid-diastole grayscale images. Panels A and B show a TEE case, and panels C and D show a CT case. For each modality, the left panel displays the result from direct registration, and the right panel shows the result from the FEM-augmented registration method. Furthermore, to assess the biomechanical behavior of different valve morphologies, we evaluated leaflet strain in adult trileaflet, adult bicuspid, and pediatric cases and compared the resulting strain ranges. The resulting strain maps exhibited physiologically consistent patterns.

### 3.1. 4D echocardiography images

#### 3.1.1. Trileaflet aortic valves

**Figure 4:**
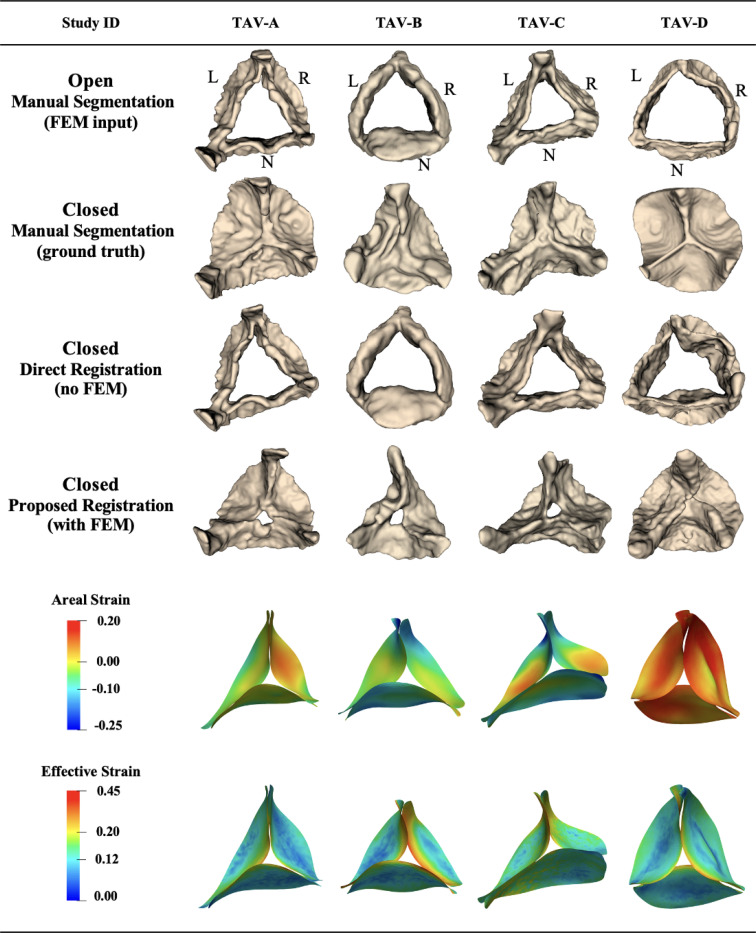
Registration and strain results for trileaflet adult aortic valves from 4D TEE images. Examples with lower (TAV-A/B/C) and higher (TAV-D) mean distance values after valve registration from open to closed configuration, computed between registered and ground-truth manual segmentations. L: left coronary leaflet; R: right coronary leaflet; N: non-coronary leaflet.

Among 14 adult 4D TEE images, 11 were trileaflet and 3 were bicuspid aortic valves. The mean distance between the propagated segmentation for the closed state and the manual ground truth segmentation for these 11 patients was 1.68 ± 0.52 mm using the proposed biomechanically informed registration approach and 2.52 ± 0.56 mm with direct registration. Figure 4 shows the propagated segmentation results and strain for four of these cases. Patients TAV-A, TAV-B, and TAV-C were selected to represent some of the best registration results, whereas patient TAV-D was selected to represent one of the worst. These four adult trileaflet valves displayed a spectrum of valve function: normal (TAV-D), mild (TAV-C), mild-to-moderate (TAV-A), and moderate (TAV-B) aortic regurgitation, with no significant stenosis. Each case originated from a distinct clinical context: aortic aneurysm (TAV-A and TAV-C), aortic valve disorder (TAV-B), and coronary artery disease (TAV-D).

The mean distance deviation from the ground truth for patients TAV-A, TAV-B, TAV-C, and TAV-D was 1.23, 1.27, 2.43, and 2.73 mm, respectively, using the proposed approach. For the same cases, the corresponding values using direct registration were 1.96, 2.08, 2.58, and 3.45 mm. Comparing the two methods, it was clear that direct registration (without FEM) could not fully reproduce leaflet closure. Among the 11 patients, one of the worst propagation results were observed for case TAV-D. Although the proposed method improved the mean distance by 21% for this case, the large and complex deformation combined with limited image quality restricted further improvement in the final registration step. The large deformation in case TAV-D could be seen by comparing the fully open and fully closed segmentations, and it was also reflected in the strain shown in Figure 4. However, the areal strain distribution was uniform, consistent with normal valve function. The maximum effective Green–Lagrange strain was observed along the coaptation lines and near the commissure points, which agreed well with previous studies (Rego et al., 2022; Abbasi and Azadani, 2015; Marom et al., 2012; Jermihov et al., 2011).

#### 3.1.2. Bicuspid aortic valves

**Figure 5:**
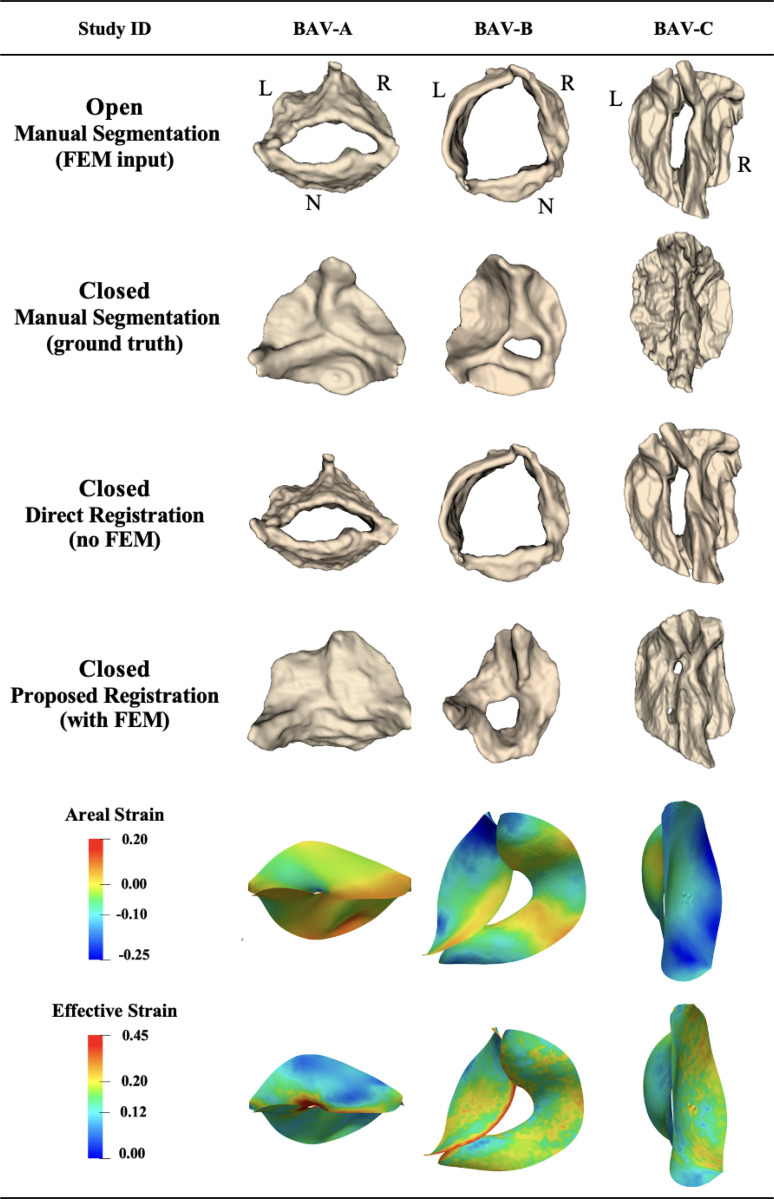
Registration and strain results on bicuspid adult aortic valves 4D TEE images, showing valve registration from open to closed configuration. L: left coronary leaflet; R: right coronary leaflet; N: non-coronary leaflet.

Among 14 adult 4D TEE images, 3 were bicuspid aortic valves. The mean distance between the propagated segmentation for the closed state and the manual ground truth segmentation for these 3 patients was 2.04 ± 0.59 mm using the proposed biomechanically informed registration approach and 3.69 ± 0.34 mm with direct registration. Figure 5 shows the propagated segmentation results and strain for these cases. These three adult bicuspid valves exhibited a range of valve function: no aortic regurgitation (BAV-C), mild regurgitation (BAV-A), and severe aortic regurgitation (BAV-B). Aortic stenosis severity varied: absent in BAV-B, mild in BAV-A, and moderate-to-severe in BAV-C. Calcification was present in BAV-C (severe). Each case arose from a distinct clinical context: ascending aortic aneurysm (BAV-A), aortic regurgitation (BAV-B), and combined aortic aneurysm and aortic valve disease (BAV-C).

The mean distance deviation from the ground truth for patients BAV-A, BAV-B, and BAV-C was 1.62, 1.78, and 2.71 mm, respectively, using the proposed approach, and these metrics for the same cases were 3.38, 3.63, and 4.06 mm using direct registration. Furthermore, we observed a link between patient physiology and strain values. Patient BAV-A had aortic valve leaflets with a larger coaptation area, which resulted in higher maximum strain (Figure 5). Patient BAV-C had severe leaflet calcification of the aortic valve, which is reflected by the lower minimal strain observed on the leaflets. Patient BAV-B had severe regurgitation, which can be observed in the propagated segmentation using proposed method in Figure 5.

### 3.2. 4D computed tomography images

**Figure 6:**
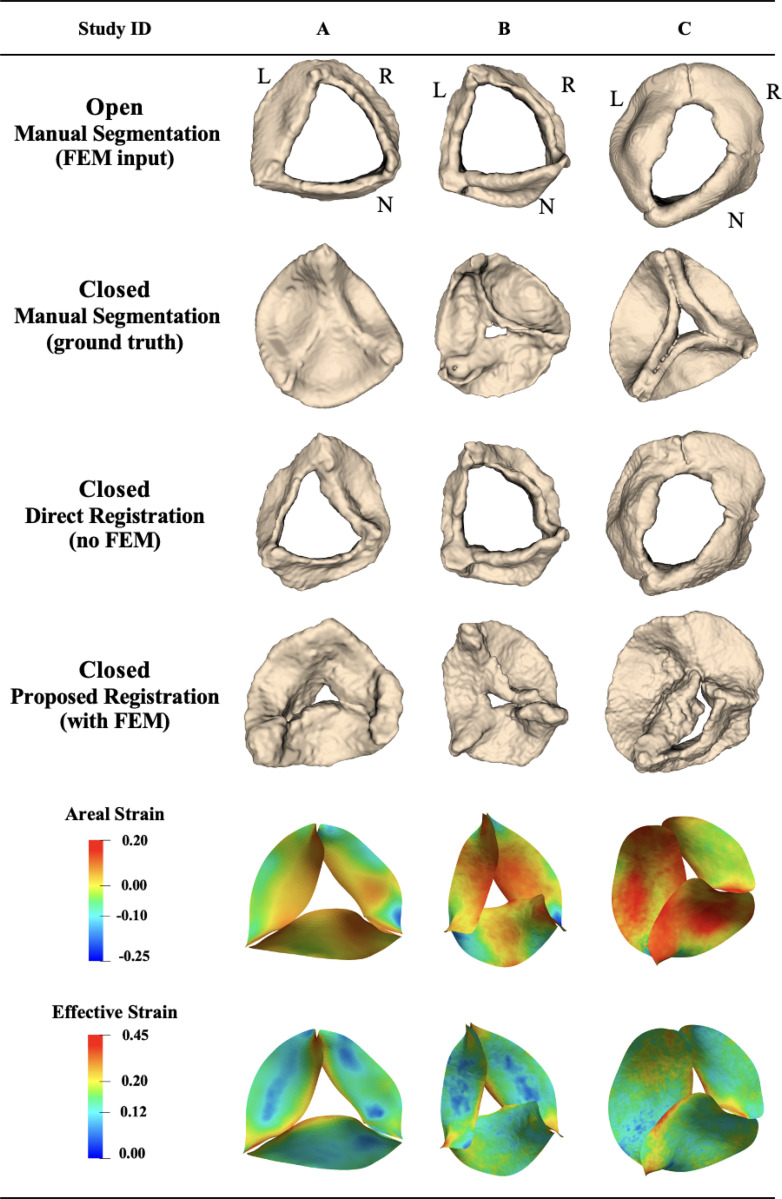
Registration and strain results for pediatric aortic valves from 4D CT images, showing valve registration from open to closed configuration. L: left coronary leaflet; R: right coronary leaflet; N: non-coronary leaflet.

The 6 patients with 4D CT images included 4 pediatric patients and 2 adults. The mean distance between the propagated segmentation for the closed state and the manual ground-truth segmentation for these 6 patients was 3.49 ± 1.68 mm using the proposed biomechanically informed registration approach and 6.44 ± 2.32 mm with direct registration. Figure 6 shows the propagated segmentation results and strain for 3 pediatric cases. Patients A and B were selected to represent some of the best registration results, whereas patient C was selected to represent one of the worst. These three pediatric aortic valves exhibited a range of function: trileaflet valves (A and B) and a unicuspid valve with partial R/N and R/L leaflet fusions (C). Aortic regurgitation severity ranged from severe (A) to moderate-to-severe (B) and moderate (C). Aortic stenosis was absent in A and B cases and mild in C. Each case arose from a distinct clinical context: severe neo-aortic insufficiency (A), moderate-to-severe neo-aortic regurgitation with moderate left ventricular dilation (B), and combined aortic valve insufficiency and stenosis (C).

The mean distance deviation from the ground truth for patients A, B, and C using the proposed approach was 1.91, 2.30, and 5.12 mm, respectively. Using direct registration, the corresponding values were 2.97, 4.50, and 9.62 mm. The worst propagation performance was observed for case C (unicuspid). In this case, the mean distance remained high for both methods, indicating inferior image quality. Although the proposed approach improved the mean distance by 47%, the large and complex leaflet deformations limited the accuracy of the FE simulation. Consequently, due to the limited image quality the final registration step also was unable to fully capture the valve motion. Overall, we observed larger deformations between the fully open and fully closed configurations in pediatric patients, particularly in case C (unicuspid), as illustrated by the manual segmentation in Figure 6. This increased deformation was further reflected in higher strain values on the valve leaflets.

### 3.3. Strain evaluation on the leaflets

**Figure 7:**
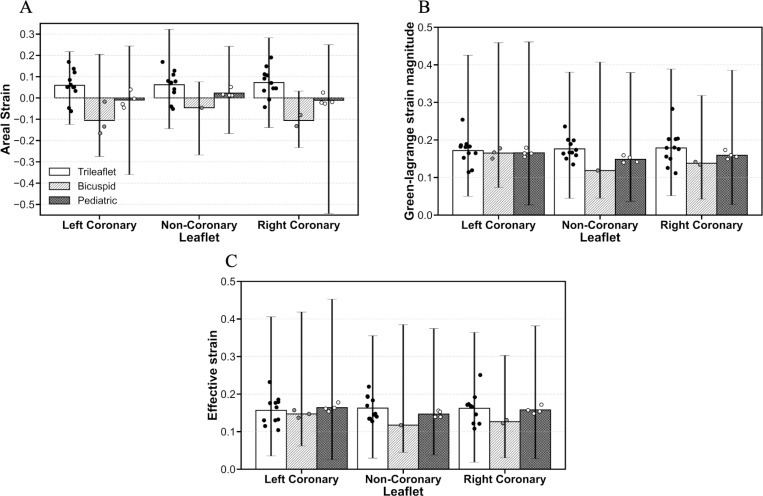
Strain analysis across the aortic valve leaflets (left coronary, non-coronary, and right coronary) in trileaflet, bicuspid, and pediatric valve groups. Bars show the mean strain for each leaflet within each valve group. Vertical ranges indicate the observed minimum–maximum strain across samples for each leaflet within each group. Individual points over each bar represent the mean strain measured on each valve leaflet. (A) Areal strain; (B) Green-Lagrange magnitude; (C) Effective strain.

Furthermore, we analyzed areal strain, the magnitude of the Green–Lagrange strain, and effective strain for each aortic valve leaflet (left coronary, non-coronary, and right coronary) in trileaflet adult, bicuspid adult, and pediatric valve groups, as shown in Figure 7. To facilitate comparison across valve groups and strain metrics, the bars in Figure 7 represent the mean strain for each leaflet within each valve group, while the vertical lines indicate the observed minimum and maximum strain for each leaflet within each group. Individual points over each bar represent the mean strain measured on each valve leaflet.

The results for areal strain across aortic valve leaflets (left coronary, non-coronary, and right coronary) in trileaflet adult, bicuspid adult, and pediatric valve groups are shown in Figure 7 A. In trileaflet adult valves, mean areal strain was 5.9%, 6.2%, and 7.2% for the left coronary, non-coronary, and right coronary leaflets, respectively. The maximum strain observed in this group was 32.2% and the minimum strain was −14.4%, both on the non-coronary leaflet. In contrast, bicuspid adult valves exhibited lower, predominantly negative areal strains: −10.6% (left coronary), −4.6% (non-coronary), and −10.6% (right coronary). The observed strain range was wide, from −27.5% to 20.5% on the left coronary leaflet, reflecting pronounced heterogeneity. Pediatric valves displayed generally lower areal strains compared with adult groups, with mean values near zero: −1.0% (left coronary), 2.3% (non-coronary), and −1.1% (right coronary). Despite these low mean values, observed strain ranges were wide, particularly for the right coronary leaflet (−54.3% to 25.0%).

The results for Green-Lagrange magnitude strain, which captures the overall local deformation including both deviatoric and volumetric components, across the same leaflets and valve groups are shown in Figure 7 B. In trileaflet adult valves, mean strains were 17.3%, 17.7%, and 17.6% for the left coronary, non-coronary, and right coronary leaflets, respectively. The maximum observed strain was 42.6% on the left coronary leaflet, and the minimum observed strain was 4.5% on the non-coronary leaflet. Bicuspid adult valves exhibited slightly lower mean strains: 16.5% (left coronary), 11.9% (non-coronary), and 13.8% (right coronary), with a maximum strain of 45.9% on the left coronary leaflet and a minimum of 4.3% on the right coronary. Pediatric valves showed mean strains comparable to adult groups: 16.6% (left coronary), 14.8% (non-coronary), and 15.9% (right coronary). Similar to areal strain, Green-Lagrange strain magnitude exhibited wide ranges, particularly for the left coronary leaflet (2.7% to 46.1%).

Finally, we present the results for effective (von Mises–type) strain, which quantifies primarily deviatoric (shape-changing) deformation, across the same leaflets and valve groups (Figure 7C). Effective strain values followed patterns similar to those observed for Green–Lagrange magnitude strain but were consistently lower, reflecting the exclusion of volumetric contributions to the total deformation. Importantly, the difference between trileaflet adult and pediatric mean strain was reduced when assessed using effective strain. Effective strain values were similar between the two groups, with trileaflet adult leaflet averages ranging from 15.9 to 16.5% and pediatric valve averages ranging from 14.7 to 16.4%, resulting in minimal group differences. This finding suggests that age and size related differences in leaflet deformation are more associated with volumetric deformation than with deviatoric deformation.

## 4. Discussion

Across 20 patients, we observed a 40% improvement in registration accuracy using the proposed FEM-augmented tracking method compared to direct registration. This improvement reflects the reduction in the mean distance between the tracked closed-state segmentation and the manual ground truth across all 20 cases, calculated as the relative decrease compared to direct registration. When analyzed separately, the improvement was 33% for the 14 4D TEE images and 46% for the 6 4D CT images, calculated in the same way for each imaging modality. The registration improvement reflects the benefit of incorporating intermediate frames from FE simulation, which were used as binary images to guide registration, whereas direct registration relies solely on image information.

Areal strain analysis revealed distinct and physiologically meaningful patterns of leaflet deformation across patient groups. Trileaflet adult valves showed positive mean areal expansion across all leaflets, consistent with symmetric valve geometry and normal physiological loading. However, individual leaflet measurements included negative values, indicating contraction even in trileaflet valves. In contrast, bicuspid adult valves exhibited overall leaflet contraction with notable asymmetry: the left and right coronary leaflets showed comparable negative strain, while the non-coronary leaflet displayed smaller contraction. Despite local leaflet expansion up to 20.5%, the overall pattern indicates restricted and uneven leaflet expansion, highlighting the mechanical consequences of fused leaflet geometry and supporting prior reports of asymmetric mechanical loading in bicuspid valves (Jermihov et al., 2011; Merritt et al., 2014; Rego et al., 2022). However, inter-leaflet variability in the bicuspid group should be made cautiously due to the limited number of available leaflets, particularly for the non-coronary leaflet. Pediatric valves showed near-zero mean areal strain but notable strain variability, particularly in the right coronary leaflet, likely reflecting ongoing growth and evolving valve geometry. The locations of extreme strain values across groups were consistent with expected deformation patterns, with higher strain near the free edge and commissures and lower strain near the annulus (Aggarwal et al., 2016).

Green–Lagrange strain magnitude analysis provided a full measure of local leaflet deformation and complemented the areal strain findings by highlighting differences in deformation across valve groups. Adult trileaflet valves exhibited a relatively uniform mean Green–Lagrange strain magnitude across all three leaflets, suggesting a balanced mechanical loading and relatively symmetric deformation under physiological conditions. Although local variability was present, strain magnitudes were broadly comparable among leaflets, consistent with structurally normal valve. Adult Bicuspid valves showed reduced mean Green–Lagrange strain magnitudes, notably on the non-coronary leaflet, reflecting the asymmetric geometry and altered load sharing associated with leaflet fusion. Although the average strains were lower, bicuspid valves exhibited some of the largest observed maximum strain magnitudes, exceeding 45% on the left coronary leaflet. This combination of reduced mean strain and elevated local maxima highlights the heterogeneous mechanical environment in bicuspid valves, where localized regions may experience substantially higher deformation despite lower overall leaflet averages. This potentially challenges tissue hemostasis and contributes to rapid disease progression. Pediatric valves demonstrated mean Green–Lagrange strain magnitudes comparable to those of trileaflet adult valves, but with greater leaflet-to-leaflet variability and broader strain ranges. In particular, the left coronary leaflet in pediatric valves exhibited strains ranging from 3% to 46%, suggesting significant strain heterogeneity. This increased variability likely reflects developmental differences in tissue structure and leaflet geometry, which may contribute to less uniform mechanical behavior during valve function. The highest Green-Lagrange strain magnitudes were observed along leaflet free edges and near commissure regions, consistent with previous studies reporting strain magnitudes from 17% to 54% (Rego et al., 2022; Abbasi and Azadani, 2015; Marom et al., 2012; Jermihov et al., 2011). Overall, these findings confirm that trileaflet adult valves exhibit relatively uniform deformation, bicuspid valves show slightly reduced and asymmetric strain patterns, and pediatric valves display more heterogeneous behavior, underscoring the importance of patient-specific mechanical assessment, particularly for valves with asymmetric or developing geometry.

Effective strain analysis showed the deviatoric (shape-changing) component of leaflet deformation. Across trileaflet adult and pediatric valves, the mean effective strains were closely aligned with one another and lower than the corresponding Green–Lagrange strain magnitudes. This convergence suggests that size and age related differences in total deformation are not primarily driven by differences in deviatoric strain, but rather by volumetric components captured in the Green–Lagrange strain. In structurally normal trileaflet valves, this indicates that the fundamental shear-dominated deformation mechanisms remain consistent across age groups despite differences in valve size.

Despite these promising results, several limitations should be acknowledged. First, the sample size was relatively small, with only 20 patients, which limits the generalizability of our findings and underscores the need for validation in larger cohorts. Second, while the proposed method improves registration accuracy, its performance is influenced by the quality of the input imaging. Third, this study focused primarily on geometric and strain-based analyses without direct correlation to long-term clinical outcomes or functional measures. Future studies are therefore needed to establish relationships between the observed mechanical patterns and clinically relevant measures. Nonetheless, the proposed framework provides an important foundation for such investigations.

## 5. Conclusion

In this study, we coupled a deformable image-based registration method with FE simulation to accurately reconstruct patient-specific aortic valve closure and perform biomechanical evaluation. This integrated framework not only improves registration accuracy but also addresses uncertainties in boundary conditions and material properties during biomechanical analysis, while reducing the reliance on fully detailed valve models that require many assumed parameters and incur high computational cost.

By accurately capturing both valve geometry and mechanics, this approach enables more reliable assessments of valve deformation patterns. Our analysis of areal strain and Green-Lagrange strain magnitude revealed that trileaflet adult valves exhibit relatively uniform deformation, bicuspid adult valves show asymmetric and locally elevated strain patterns, and pediatric valves display the greatest areal strain variability across leaflets. These findings emphasize the heterogeneity of valve mechanics across different patient populations. Furthermore, the convergence of mean effective strain between trileaflet adult and pediatric valves indicates that age and size related differences in total deformation are not primarily driven by deviatoric (shape-changing) strain, but rather by volumetric deformation components captured by the Green-Lagrange strain magnitude.

Importantly, the detailed strain distributions obtained through this method can provide potential clinical insights: regions of abnormally high or low strain may indicate a higher risk of tissue remodeling, dysfunction, or failure, which could guide repair strategies and risk assessment. Therefore, this framework supports the development of individualized therapeutic strategies and advances our understanding of patient-specific valve behavior.

## Figures and Tables

**Table 1:** Patient characteristics aortic valve

Characteristic	Adult tricuspid (n = 13)	Adult bicuspid (n = 3)	Pediatric (n = 4)
**Morphology**	Trileaflet	Bicuspid (variable fusion patterns)	Trileaflet (n = 3), Unicuspid (n = 1; partial fusion)

**Regurgitation severity**	None: 6 Mild: 2 Moderate: 2 Moderate-to-severe: 1 Severe: 2	None: 1 Mild: 1 Severe: 1	Moderate: 1 Moderate-to-severe: 1 Severe: 2

**Stenosis severity**	None: 13	None: 1 Mild: 1	None: 3 Mild: 1
		Moderate-to-severe: 1 (calcification)	
